# Multiregional Population Forecasting: A Unifying Probabilistic Approach for Modelling the Components of Change

**DOI:** 10.1007/s10680-025-09729-7

**Published:** 2025-04-10

**Authors:** Arkadiusz Wiśniowski, James Raymer

**Affiliations:** 1 Social Statistics Department, University of Manchester, Oxford Rd, Manchester, M13 9PL UK; 2 School of Demography, Australian National University, 146 Ellery Crescent, Acton, ACT 2601 Australia

**Keywords:** Demographic forecasting, Multiregional demography, Projections, Bayesian inference, Australia

## Abstract

In this article, we extend the multiregional cohort-component population projection model developed by Andrei Rogers and colleagues in the 1960s and 1970s to be fully probabilistic. The projections are based on forecasts of age-, sex- and region-specific fertility, mortality, interregional migration, immigration and emigration. The approach is unified by forecasting each demographic component of change by using a combination of log-linear models with bilinear terms. This research contributes to the literature by providing a flexible statistical modelling framework capable of incorporating the high dimensionality of the demographic components over time. The models also account for correlations across age, sex, regions and time. The result is a consistent and robust modelling platform for forecasting subnational populations with measures of uncertainty. We apply the model to forecast population for eight states and territories in Australia.

## Introduction

Regional population forecasts are important for planning and understanding how populations are changing and redistributing. To forecast regional population changes, one must have a mechanism to capture different sources of population growth. In low fertility and developed societies, the main factors driving population redistribution are internal migration and immigration, for which both tend to concentrate people towards large metropolitan areas. Forecasting regional populations interconnected by internal migration is challenging because, to have consistency, the model should include origin–destination matrices of age-specific migration transition probabilities (Rogers, 1995). This means that for a set of *n* regions, one must model *n* by $n-1$ transition probabilities which may or may not be correlated with each other.

In addition to the need for robust subnational population projections, there is the need for information regarding their relative quality. While some developments have been made to address the challenges for probabilistic multiregional population estimation and projections (Bryant & Graham, 2013; Gullickson & Moen, 2001; Raymer et al., 2012; Sweeney & Konty, 2002; Wilson & Bell, 2007), a unifying probabilistic framework has yet to come together. The aim of this paper is to provide such a framework based on recent developments in Bayesian population forecasting (Wiśniowski et al., 2015). Bayesian inference allows combining expert opinions and knowledge about demographic processes with empirical data to form probabilistic estimates and forecasts. This differs from purely data-driven frequentist approaches (e.g. Hyndman & Booth, 2008). Traditionally, Bayesian inference was not widely used due to difficulties in computation but, more recently, with enhanced computing power, there have been numerous applications in demography (see, e.g., Bijak & Bryant, 2016) and across the social sciences (e.g. Jackman, 2000; Lynch & Bartlett, 2019).

The main principles motivating this paper and the models developed are: (i) the demographic components are modelled by using their cross-tabulations at the age, sex and region level; (ii) populations interact through internal migration; and (iii) measures of uncertainty are included so that the likelihood of future population change is better understood. This research substantially extends earlier efforts in regional population estimation and projection by the inclusion of probabilistic information within the multiregional population projection model framework. Despite fifty years of development and evidence for more accurate and less biased projections, multiregional projection models are still under-utilised by national statistical offices and the production of probabilistic forecasts is largely non-existent. Instead, national statistical offices tend to rely on relatively simple deterministic assumptions regarding net migration or gross flows of in-migration and out-migration that are often held constant for the foreseeable future (Cappelen et al., 2015). These models do not take into account the linkages between origins and destinations and often have to be adjusted to ensure net migration summed across regions is zero. This is concerning since both internal migration and international migration are increasingly becoming the dominant sources of demographic change. Moreover, many developed countries have high-quality data on these components, but they are not fully utilised to improve estimation and prediction of subnational population change.

The modelling framework developed in this article integrates forecasts of age-, region- and sex-specific births (by age of mother), deaths, internal migration and international migration for subnational populations in Australia. The framework utilises Bayesian inference and is motivated by the ideas developed by Wiśniowski et al. (2015) for national-level forecasting. Here, we propose combining log-linear models, which capture key structures in contingency tables, with bilinear models widely used to forecast age patterns of demographic components. This combination provides a parsimonious and flexible model specification that can be applied to all population components of change.

## Background

Multi-state or multiregional population models may be considered extensions of the life table and the cohort-component projection model. These models allow populations to move between various states in their life course, providing the analyst with a means to better model and understand the mechanisms underlying population change. The life course transitions may include those between states of residences, employment, marriage or health. Early developments of this modelling framework can be found in Rogers (1975), Land and Rogers (1982) and Schoen (1988). Term “multi-state” refers to a more general form of population modelling, whereas “multiregional” refers specifically to the inclusion of origin–destination-specific migration rates or probabilities (Rogers, 1975, 1995). Hereafter, we focus on multiregional population projections.

Multiregional population models provide a general and flexible platform for modelling and analysing subnational population changes over time. However, while there are many examples of multiregional or multistate population models applied to study population change from which we can draw experience (e.g. Espenshade, 1983; Rees, 1986b; Rees & Willekens, 1989; Rogers et al., 1999; Rogers & Raymer, 1999; 2001; Rogers, 2015; Rogers et al., 1989; Rogers & Willekens, 1986; Willekens, 1980; Willekens et al., 1982), including analyses focused on Australia (Wilson, 2009; Wilson & Bell, 2004; Raymer et al., 2020a), very little research has been carried out in the area of probabilistic multiregional forecasting (see also discussions in Wilson & Rees, 2005; Wilson & Bell, 2007). The exceptions are Rees and Turton (1998) and Gullickson (2001).

Most standard (uniregional) cohort-component population projection models ignore migration transitions and instead rely on net migration or (slightly better) out-migration and in-migration^1^ rates to account for the change due to migration. The problem with net migration and in-migration rates is that they include the incorrect population at risk of migrating in the denominator, which can seriously bias the results (Rogers, 1990) and uncertainty measures (Raymer et al., 2012). Moreover, as mentioned previously, most national statistical agencies choose to rely on relatively simple accounting models to produce estimates of population by age, sex and region, which do not include uncertainty. Considering the wide usage of national statistics projections, and the amount of resources distributed based on them, improved methods to forecast subnational populations and their compositions are important for the user community.

In this article, we propose a probabilistic framework for forecasting the regional components of change and for the dynamic modelling of subnational populations. To achieve this aim, we combine multiregional life tables and projections (Rogers, 1995; Schoen, 2007) and recent advances in probabilistic forecasting of demographic components (Wiśniowski et al., 2015). With the combination of empirical data, statistical modelling techniques and the knowledge of demographic behaviours, this research advances the building and application of dynamic population models.

## Modelling Framework

### Multiregional Cohort-Component Projection Model

In our application, we rely on the classical specification of the multiregional projection model for an open population as described by Rogers (1995) for five-year age groups. In our illustration of the method using data for Australia, the baseline year for the forecasts is 2011 and we forecast three five-year periods until the year 2026. The available data on internal migration represent 5-year transitions between places of residence. Thus, the approach taken is the “Option 2” approach for the transition data (Rogers, 1995, p. 97). The movement or migration event data approach (“Option 1”) is described in Appendix A. Note the methods can be readily adapted for single years of age if such data are available.

Let $K_{x}(t)$ denote a vector of the population aged *x* to $x+4$ in year (*t*) that contains concatenated sub-populations of males (*M*) and females (*F*) in each region *r*. We project the population for all age groups *x* in year $(t+5)$ to be^2^:

1 $$\begin{aligned} K_0(t+5)&=\frac{5}{2}\textbf{S}_{-5}\sum _{x=\alpha }^{\beta }\left( B_{x}+ \textbf{S}_{x}B_{x+5}\right) \left( K_{xF}\left( t\right) +\frac{1}{2}G_{xF}\left( t\right) \right) +\frac{1}{2}G_{0}\left( t\right), \end{aligned}$$

2 $$\begin{aligned} K_{x+5}\left( t+5\right)&=\textbf{S}_{x}\left( K_{x}\left( t\right) +\frac{1}{2}G_{x}\left( t\right) \right) +\frac{1}{2}G_{x+5}\left( t\right) , \end{aligned}$$

where *B* denotes a vector of age-specific fertility rates for all regions and applicable only to the female population, $\alpha$ and $\beta$ denote the first and last reproductive age groups, respectively (15–19 and 45–49 in our application), and *G* denotes a vector of total immigrants projected to arrive during each 5-year period by age, sex and region. The births and immigrants contributing to the 0–4-year-old population, $K_0$, are then split into males and females assuming there are 105 males for every 100 females. The survivorship matrix $\textbf{S}$ is derived from age-specific probabilities of interregional migration, mortality and emigration (Rogers, 1995, p. 101):

3 $$\begin{aligned} \textbf{S}_x = \left( \textbf{I}+ \textbf{P}_{x+5}\right) \textbf{P}_x\left( \textbf{I}+\textbf{P}_x\right) ^{-1}, \qquad x=0,5,\ldots ,z-5, \end{aligned}$$

where $\textbf{I}$ denotes an identity matrix, $\textbf{P}_x$ and $\textbf{P}_{x+5}$ are age-specific matrices that specify probabilities of interregional migration, mortality and emigration, and *z* is an open-ended terminal age group (in our illustration it is 85+). $\textbf{P}_x$ is derived using linear interpolation (Equation 4.26 in Rogers, 1995):

4 $$\begin{aligned} \textbf{P}_{x}&=\overline{\textbf{P}}_{x}\ \textbf{P}_{x}^{DE}, \qquad \forall x, \end{aligned}$$

5 $$\begin{aligned} \overline{\textbf{P}}_{x}&=\frac{1}{2}\left( \overline{\textbf{S}}_{x}+\overline{\textbf{S}}_{x-5}\right) ,\qquad x=5,10,\ldots ,z, \end{aligned}$$

with $\overline{\textbf{S}}_x$ being a matrix of conditional survivorship proportions with elements $m_{ijx}$ calculated as proportions of out-migration from region *i* to region *j*, by comparing residences at the beginning and end of the period. Conditioning is on the fact that these proportions do not account for mortality and emigration. $\overline{\textbf{P}}_x$ is a matrix of conditional transition probabilities comprised of destination-specific out-migration. For the youngest age group, we use an approximation $\overline{\textbf{P}}_0=\frac{1}{2}\left[ (\overline{\textbf{S}}_0)^2+\overline{\textbf{S}}_0\right]$ and assume $\overline{\textbf{P}}_z=\overline{\textbf{S}}_z$ for the oldest age group (Rogers, 1995, p. 98). The diagonal matrix $\textbf{P}_x^{DE}$ “un-conditions” the transition probabilities and is constructed for each region *r* using rates of mortality ($d_{rx}$) and emigration ($e_{rx}$):

6 $$\begin{aligned} P_{rr,x}^{DE}&=\frac{1-\frac{5}{2}\left( d_{rx} + e_{rx}\right) }{1+\frac{5}{2} \sum _{k=1}^{n}\overline{P}_{rk,x}\left( d_{kx}+ e_{kx}\right) }\qquad \forall x,r, \end{aligned}$$

with $\overline{P}_{rk,x}$ being elements of matrix $\overline{\textbf{P}}_x$. The inclusion of immigration counts (*G*) recognises the absence of a well-defined population at risk, whereas for emigration (*e*) it makes sense to use rates because there is a clear population at risk (Rees, 1986a). Finally, the survivorship of 0–4-year-olds is calculated using the approximation derived by Rogers and Ledent (1976) and Ledent (1978, pp. 48–49) that uses a matrix of mortality rates for the first age group, $\textbf{M}_0$:

7 $$\begin{aligned} \textbf{S}_{-5}&=\left( \textbf{I}+\frac{5}{2}\textbf{M}_{0}\right) ^{-1}=\left[ \textbf{I}+(\textbf{I}+\textbf{P}_0 )^{-1} \left( \textbf{I}-\textbf{P}_0 \right) \right] ^{-1}. \end{aligned}$$

**Fig. 1:**
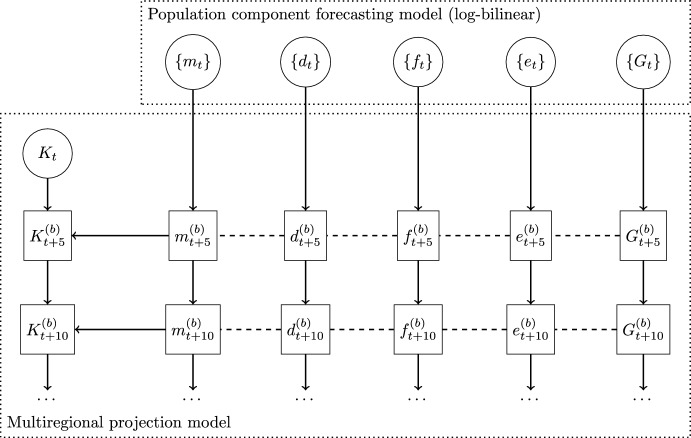
Conceptual representation of the modelling framework. Circles are input data; squares are estimates with (*b*) denoting *b*th sample from the probability distribution; *K* is population; $\{\cdot \}$ denotes time series of population components data: *m*—internal migration, *d*—mortality rates, *f*—fertility rates, *e*—emigration rates, *G*—immigration counts.

To produce quinquennial probabilistic forecasts of $K(t+5)$, the projection model requires as input a baseline population *K*(*t*), and predictive probability distributions for: (i) out-migration probabilities $m_{ijxs}(t+5)$, (ii) mortality rates $d_{rxs}(t+5)$, (iii) fertility rates (applied to female population) $f_{rx}(t+5)$, (iv) emigration rates $e_{rxs}(t+5)$, and (v) immigration counts $G_{rxs}(t+5)$. A probability distribution of forecasted population is created by updating a baseline population using the projection model (1 and 2) with samples from the predictive distributions of population components produced using log-bilinear models (Sect. 3.2) as presented in Fig. 1. This framework accounts for uncertainty about the rates of population components, but not the uncertainty about the future counts; in other words, the Poisson variability used in log-bilinear models is not incorporated in the forecasts. This is a standard practice in demographic forecasting, also at subregional level (Yu et al., 2023; Wiśniowski and Raymer, 2016). Here and in our illustration using Australian data and similarly to Yu et al. (2023), we also assume the baseline population is true (without error) but, in principle, a probability distribution could be included (Wheldon et al., 2013).

The specification of the above projection model assumes the following. First, survivorship and birth rates are specific to the region where deaths, emigration or births occur, which may be not exactly the same as the group of population that experiences them (due to migration to and from the regions). Second, in the application, we use 18 five-year age groups from 0–4 to 85+ years. This relatively broad age grouping may mask important age-specific demographic developments occurring within each age group, especially in the open-ended last age group. Third, simple linear approximations are used to estimate proportions of person-years lived in each period (Rogers, 1975, p. 66), which again may exclude important patterns in the youngest and the oldest age groups. To estimate survivorship of the oldest population more precisely, the age groups could be extended by applying, for example, graduation techniques (Preston et al., 2000; Dodd et al., 2018).

### Models for Forecasting Demographic Components of Change

The models for forecasting the demographic components of change (rates and counts, as shown in Fig. 1) developed for this research are based on the combination of multiplicative component or log-linear models (e.g. Stillwell, 1986; van Imhoff et al., 1997; Van der Gaag et al., 2000; Raymer et al., 2006; van Wissen et al., 2008; Raymer et al., 2020b) and bilinear models (e.g. Lee & Carter, 1992; Lee, 1993; Booth & Tickle, 2008; Hyndman & Booth, 2008). They also represent an extension of the methods implemented in Wiśniowski et al. (2015) to forecast the total population of the UK by age and sex.

The general approach to building a multiregional projection model includes decomposing a high dimensional array of population data cross-classified by region, age, sex and time data into lower (single- or two-) dimensional arrays, such as region by age, region by sex or region by time. For instance, for internal migration count data $Y_{ijx}$ cross-classified by the $i=1,\ldots ,n$ regions of origin (subscript *i*), $j=1,\ldots ,n$ regions of destination (*j*) and $x=1,\ldots ,z$ age groups, we can write a model:

8 $$\begin{aligned} Y_{ijx} = c\times O_i\times D_j\times A_x, \end{aligned}$$

where *c* is a scalar constant, and *O*, *D* and *A* are vector parameters (main effects) of lengths *n*, *n* and *z*, respectively, capturing the overall level of out-migration (*c*) and the proportions of migration from each origin (*O*), to each destination (*D*) and in each age group (*A*). In the frequentist approach, the log-linear models can be estimated within a Generalised Linear Model (GLM) by assuming that the count data *Y* are Poisson distributed with a logarithmic link function and a linear predictor being formed by logarithms of the multiplicative effects (*O*, *D*, etc). For an introduction to log-linear models, refer to Agresti (2013), and for applications in forecasting internal migration, refer to Willekens and Baydar (1986).

The above model can be extended to include two-way interaction terms by adding a vector parameter of size $n^2$ that captures the interactions between places of origin and places of destination:

9 $$\begin{aligned} Y_{ijx} = c\times O_i\times D_j\times A_x\times OD_{ij}. \end{aligned}$$

This specification allows for more control of the forecasting process, where for example, the overall level (*c*) and age main effect parameters (*A*) vary over time, but the parameters for the origin main effects, destination main effects and origin–destination interactions are held constant. A wide range of other options could be tested both with various configurations of model parameters and time-varying aspects, before settling on the most suitable forecasting model.

One can select the final model based on goodness of fit to the data, cross-validation (i.e. by using training and test sub-samples of data) or information criteria (Raymer et al., 2020b). The number of parameters in a log-linear model for a multi-dimensional contingency table can be large (e.g. a model with all possible interactions has as many parameters as there are observations). Hence, in our approach, we select the final model that is used to forecast a given population component by, firstly, simplifying the models into log-linear form without a bilinear term and, secondly, selecting a model that yields the smallest root mean square error (RMSE) of the residuals. We also consider Schwarz (Bayes) Information Criterion, which favours parsimonious models (Upton, 1991).

Next, we propose that the main effect and interaction terms are forecasted by integrating a time series component in the model. Our approach is similar to the bilinear model proposed by Lee and Carter (1992) to forecast age-specific mortality. The Lee–Carter model can be written as

10 $$\begin{aligned} \log Y_{xt}=\alpha _x + \beta _x\kappa _t +\xi _{xt},\qquad \xi _{xt}\sim \textrm{Normal}(0,\sigma ^2), \end{aligned}$$

where $\log Y_{xt}$ are logged mortality rates, which are decomposed in the model into the age schedule averaged over time (parameter $\alpha _x$), average changes of that schedule over time ($\beta _x$), with the size of changes over time captured by time parameter $\kappa _t$. A random walk with drift model is then used to forecast $\kappa _t$. The method has been greatly extended to adopt it to various data settings and population components (e.g. Booth & Tickle, 2008; Hyndman & Ullah, 2007; Antonio et al., 2015; Wiśniowski et al., 2015). The approach is flexible in the sense that it accommodates forecasting of both probabilities (or rates) and counts, subject to identifiability constraints placed on the model parameters.

For our demographic component forecasting models, we assume that counts of region-, age-, sex- and time-specific events $Y_{rxst}$ (for internal migration, subscript *r* is replaced with *ij* - for origin and destination) are Poisson distributed:

11 $$\begin{aligned} Y_{rxst}&\sim \textrm{Poisson}\left( \mu _{rxst} K_{rxst}\right) , \end{aligned}$$

where $\mu$ denotes either a probability (for internal out-migration *m* in Fig. 1), a rate (mortality *d*, emigration *e*, and fertility *f*), or counts (immigration *G*) of the demographic event and *K* is exposure or denominator (the population at risk of experiencing those events, or offset; see Agresti, 2013).^3^ This is similar to the assumption used in the GLM estimation framework. In our approach, we further assume that the logarithm of $\log \mu$ is normally distributed:

12 $$\begin{aligned} \log \mu _{rxst}&\sim \textrm{Normal}\left( \mathcal {M}, \sigma ^2\right) , \end{aligned}$$

where mean $\mathcal {M}$ denotes the expectation and $\sigma ^2$ is a variance, which allows over-dispersion in excess of the Poisson variability in (11). We assume that $\mathcal {M}$ takes the form of a log-linear model with a bilinear term, hereinafter called log-bilinear. We illustrate the construction of all component-specific models with data for Australia.

Details of the model selection for each component are presented in Appendix C. In our illustration, all selected models contain bilinear terms for age and region (except for the model for mortality), as explained in the reminder of this section. The time effects, $\kappa _t$, are forecasted using a time series model. In our illustration with data for Australia, we rely on univariate and multivariate stationary autoregressive (AR(1)) and random walk (RW) models. For instance, a stationary univariate AR(1) model is

13 $$\begin{aligned} \kappa _t&\sim \textrm{Normal} \left( \phi _1+\phi _2 \kappa _{t-1},\sigma _{\kappa }^2 \right) ,\qquad \phi _2\in (-1,1). \end{aligned}$$

With $\phi _2=1$, (13) becomes a random walk with drift model. Wiśniowski et al. (2015) and Raymer and Wiśniowski (2018) provide examples of this approach in forecasting age profiles of national-level demographic components, including age- and sex-specific flows of immigration and emigration.

The time series models used in this research are capable of capturing correlations between observations over time and, in the multivariate case, between population components (e.g. between emigration and immigration). The assumption of stationarity in the models implies that the uncertainty of the forecasts is finite in the long term. This uncertainty can, however, become very large, depending on variability and length of the series at hand, and assumed prior distributions. A random walk specification, on the other hand, leads to an ever increasing forecast uncertainty. Thus, we prefer the stationarity assumption for our short- and medium-term forecasts. For long-term forecasts (e.g. similar to the forecasts produced by the UN, see Azose et al., 2016), expert opinion might be incorporated to inform variance parameters (e.g. Billari et al., 2014).

To estimate model parameters, we utilise Bayesian inference. All unknown parameters are treated as random variables that have probability distributions. Computations for our models presented in this paper were carried out using Hamiltonian Monte Carlo (HMC) and No-U-Turn Sampler, implemented in R 3.6.0 (R Core Team, 2019) using rstan package for Bayesian inference (Hoffman and Gelman, 2014; Carpenter et al., 2017; Stan Development Team and others, 2018). Code and data required for computations are available in open access repository available at 10.5281/zenodo.14680112.

#### Interregional Migration

To forecast patterns of internal migration between regions, (11) and (12) become $Y_{ijxst}\sim \textrm{Poisson}\left( \mu _{ijxst} K_{ixs(t-2.5)}\right)$ and $\log \mu _{ijxst} \sim \textrm{Normal}(M, \sigma ^2)$, respectively. Exposure $K_{ixs(t-2.5)}$ represents the population at risk of transition in region *i* five years earlier.^4^ In this case, the $\mu$ parameters can be interpreted as out-migration probabilities for all origin–destination-specific flows.

For the Australian data, we consider the following specification of model $\mathcal {M}$^5^:

14 $$\begin{aligned} \mathcal {M}&= c + AS + OA + DA + OD_1 + OD_2 \kappa _{1t} + A_1 + A_2 \kappa _{2t}, \end{aligned}$$

where *c* denotes an overall intercept (i.e. the average log-rate or log-count over all years, ages, sexes, origins and destinations), *AS*, *OA* and *DA* represent two-way interactions, on a logarithmic scale for sex–age, origin–age and destination–age. All elements of these two-way interaction parameters are estimable; thus, there is no need for including main effects, such as *A* or *S*. Parameter $OD_1$ captures average origin and destination ‘profile’, and $OD_2$ is a parameter reflecting the change of origin–destination profile over time in response to time effect $\kappa _{1t}$. Analogously, $A_1$ captures the average profile of age-specific out-migration probabilities (it is an analogue of $\alpha _x$ in Eq. 10), $A_2$ the changes of that profile over time ($\beta _x$ in Eq. 10) in response to time effect $\kappa _{2t}$.

In the model for internal migration, we assume that migration from region *i* to region *j* is correlated with migration in opposite direction, from *j* to *i*.^6^ We achieve that by assuming that

15 $$\begin{aligned} OD_{1ij}&\sim \textrm{Normal}\left( \alpha _{ij}, \sigma _{OD}^2\right) , \end{aligned}$$

16 $$\begin{aligned} \alpha _{ij}&= \alpha _{ji}, \qquad \forall i,j,i\ne j, \end{aligned}$$

and $\sigma _{OD}^2$ is a scalar variance parameter.

To forecast $\kappa _{1t}$ and $\kappa _{2t}$ we use AR(1) model with drift as specified in (13). The log-bilinear model for internal migration captures the main characteristics underlying the patterns, that is, differences between sexes age profiles, origins, destinations and ages, as well as patterns over time in the origin–destination flows and age profiles (term *ODT* in Model m8 improves fit considerably over all Models m1-m7, including model with three-way interaction *AST*, and is more parsimonious than Model m10, see Table 1). In other contexts and countries, where differences between males and females are larger, additional interactions, such as *OS*, *DS* or *OSA* and *DSA*, could be tested and included in the forecasting model.

#### Mortality

To produce forecasts of mortality rates *d*, the exposure $K_{rxst}$ is a mid-year population in which deaths occur. For model $\mathcal {M}$, we assume:

17 $$\begin{aligned} \mathcal {M} = c + RA + RS + AS_1 + AS_2 \kappa _{st}, \end{aligned}$$

where *RA* and *RS* denote region–age and region–sex-specific interactions, and $AS_1$ and $AS_2$ capture the average age profiles of mortality for males and females, and changes in their profiles over time, respectively. Parameters $\kappa _{st}$ capture the time pattern of these changes over time for males ($s=M$) and females ($s=F$).

The above model for mortality (Model m8 in Table 2) does not include the region–time bilinear term. Although Model m9 with the region–time interaction produced the lowest RMSE, its inclusion led to unlikely mortality forecasts that included some life expectancy values decreasing across regions. We also did not entertain Model m6 with the lowest BIC as it did not include separate development of mortality curves over time for males and females. The model in Eq. (17) implies changes in the rates occur at the same pace in all regions but may differ between males and females. The two-way interaction terms, *RA* and *RS*, ensure that the regional differences amongst age groups and between sexes, respectively, are reflected in the forecasts. These differences are, thus, assumed to remain constant over time.

To forecast $\kappa _{st}$ we use the following multivariate vector random walk model:

18 $$\begin{aligned} \left( \begin{array}{c} \kappa _{Mt}\\ \kappa _{Ft} \end{array} \right)&\sim \mathrm {Multivariate\ Normal}\left[ \left( \begin{array}{c} \phi _{11}+\kappa _{M\ t-1}\\ \phi _{12}+\kappa _{F\ t-1} \end{array} \right) ,\mathbf {\Sigma _1}\right] , \end{aligned}$$

where $\mathbf {\Sigma _1}$ is a covariance matrix that can be decomposed as

19 $$\begin{aligned} \mathbf {\Sigma _1}&=\textbf{D}^{-1}\mathbf {\Omega } \textbf{D}^{-1}, \end{aligned}$$

where $\mathbf {\Omega }$ is a correlation matrix and $\textbf{D}=\sqrt{\textrm{diag}(\mathbf {\Sigma _1})}$ is a diagonal matrix containing square roots of the diagonal elements of $\mathbf {\Sigma _1}$, denoted further as $\sigma _M$ and $\sigma _F$ (i.e. marginal standard deviations). The model thus captures correlations between male and female mortality improvements (Table 3).

This specification is similar to coherent mortality forecasts which assume that mortality patterns for various regions do not diverge over a long period of time (Li & Lee, 2005). Li and Lee (2005) use an average region-specific age profile ($AR_1$ rather than $AS_1$ in Eq. 17), with common changes of mortality ($A_2$) and common drift parameter (i.e. $\phi _{11}=\phi _{12}$ in Eq. 18). Our proposed specification can be extended to include a rotation method (Li et al., 2013; Vékás, 2020) that adjusts changes in age patterns over time ($AS_2$) to gradually decelerate the decline of mortality at younger ages and accelerate them at older ages. Also, changes to age patterns over time can be captured by using principal components analysis with two or more principal components included in the model (Hyndman & Ullah, 2007; Antonio et al., 2015; Alexander et al., 2017).

#### Fertility

For fertility, we specify the model $\mathcal {M}$ to reflect the varying patterns of fertility across eight states or territories of Australia (Fig. 3). This model is specified as:

20 $$\begin{aligned} \mathcal {M}&= c + RA + A_1 + A_2 \kappa _{1t} + R_1 + R_2\kappa _{2t}, \end{aligned}$$

where *RA* denotes region–age-specific interactions capturing changes in age profiles of fertility in various regions, $A_1$ and $A_2$ capture the average age profile of fertility and changes over time in this profile, respectively, $R_1$ and $R_2$ capture regional average profile and its changes over time, respectively, and $\kappa _{1t}$ and $\kappa _{2t}$ represent the time effects for age and region profiles, respectively. The exposure in (11) is $K_{rxFt}$, that is, a mid-year population of females for all *r*, *t* and $\alpha \le x\le \beta$ (cf. Eq. 1).

To forecast parameters $\kappa _{1t}$ and $\kappa _{2t}$, we use univariate AR(1) models without drift, that is, $\kappa _t \sim \textrm{Normal} \left( \phi _{21} \kappa _{1\ t-1}, \sigma _{\kappa }^2\right)$. The forecasts of the TFR based on this model remain relatively constant over time with uncertainty reflecting the variability in the historical data. A random walk model may also be used (see TFR for Australia in Fig. 3), but we found the forecast uncertainty resulting from the AR(1) models to be more plausible.

The absence of drift parameters in the time series specification of the fertility model prevents the forecasts from unrealistically increasing or decreasing over time. The model captures the variability in the data on the age-specific rates over time, which, in Australia, can be attributed to postponement of childbearing (cf. Lattimore & Pobke, 2008) related to increasing educational attainment by women (Lazzari, 2019). In this situation, the inclusion of a long-term trend is likely to result in implausible fertility rates (Sobotka, 2017). If short-term forecasts are required, then localised trend time series approaches could be included and applied (e.g. Bryant & Zhang, 2018). Alternatively, two principal components can be used to capture the postponement effect (cf. Vanella and Deschermeier, 2019).

#### Immigration and Emigration

For forecasting international migration to and from Australia, we added a parameter to capture the change in the definition of migration in 2006 that was implemented since 2004 (Temple & McDonald, 2018). Our preliminary tests of goodness of fit showed that the region–sex interaction was not improving fit (Table 4 and Appendix C; Models m4-6 show that the RMSE reduces only slightly with an *RS* term). We thus assume that, for immigration, the logarithm of counts follows normal distribution with expectation

21 $${\mathcal{M}} = c + R_{{1(t \ge 2004)}} + RA + AS_{1} + AS_{2} \kappa _{{1st}} + R_{1} + R_{2} \kappa _{{2t}}$$

where $1(t \ge 2004)$ denotes an indicator taking value 1 if the time index represents year 2004 or later. For emigration rates, the model is virtually the same (cf. Table 5) but here, $K_{rxst}$ represents the exposure, i.e. the mid-year population in year *t*, region *r*, age *x* and sex *s*. In the case of immigration, we model counts rather than rates, thus exposure $K_{rxst}\equiv 1$ for all *r*, *x*, *s* and *t*.

To forecast immigration counts and emigration rates, we utilise the vector autoregressive model (VAR(1)) to capture correlations between male and female migration and constrain the patterns to not exhibit explosive behaviour over time:

22 $$\begin{aligned} \left( \begin{array}{c} \kappa _{1Mt}\\ \kappa _{1Ft} \end{array} \right)&\sim \mathrm {Multivariate\ Normal}\left[ \left( \begin{array}{c} \phi _{11}+\phi _{21}\kappa _{1M\ t-1}\\ \phi _{12}+\phi _{22}\kappa _{1F\ t-1} \end{array} \right) ,\mathbf {\Sigma _2}\right] , \end{aligned}$$

with $\mathbf {\Sigma _2}$ constructed in a similar fashion as for mortality in (19). The regional time effects both in immigration and emigration model, $\kappa _{2t}$, follow the same univariate AR(1) process. In practice, the stationarity assumption can be violated and migration has exhibited highly volatile patterns in response to economic, political, or environmental shocks (cf. Bijak et al., 2019). However, similar to the fertility forecasting model, the posterior distributions of the autoregressive parameters can take values in ranges very close to unity yielding forecasts with (almost) ever increasing uncertainty that stabilises only in very distant horizons. Also, a stationary specification “protects” the forecasts against exhibiting explosive and thus unrealistic patterns.

In principle, we can model immigration and emigration together, for example, by using a four-dimensional multivariate model. However, as explained in Sect. 3.1, the adopted multiregional projection model specification requires immigration counts and emigration rates as inputs. While emigration and immigration counts (rates) are usually highly correlated, the inclusion of correlations between emigration rates and immigration counts would misrepresent the variability in the data. For example, when the population of interest is relatively small, it can be significantly affected by high volumes of emigration and/or immigration. Stable counts of emigration over time would imply increasing rates if not counter-balanced with corresponding counts of immigration.

### Prior Distributions and Parameter Identification

In our application, we assume weakly informative prior distributions for all model parameters. This assumption allows the likelihood to dominate the posterior distribution where abundant (and presumably accurate) data are available. In general, one could specify priors by using prior predictive distributions for the data and assessing if the implied ranges of interpretable parameter values are demographically plausible (Gabry et al., 2019, p.393). Further, overly vague priors may lead to numerical instability and problems with identification of log-linear model parameters, which are naturally correlated with each other. For instance, main effects for age (*A*) describe average age profile. For these parameters, we assume a prior that is wider than the prior for the two-way region–age (*RA*) interaction, which is here interpreted as a region–age-specific deviation from that main profile *A*.

In our framework, the main effects parameters, such as $A_1$ and $R_1$, are assumed to be normally distributed with means set to zero and standard deviations with half-*t* distributions:

23 $$\begin{aligned} A_1&\sim \textrm{Normal} \left( 0, \sigma _A^2\right) ,&\sigma _A \sim \textrm{t}_+ \left( 0, 2.5,0.5\right) , \end{aligned}$$

where $\textrm{t}(\mu ,\nu ,\sigma )$ denotes a Student-*t* distribution with degrees of freedom $\nu$, location parameter $\mu$, and scale parameter $\sigma$.^7^ The same prior is assumed for $\sigma$ in Eq. (12).

The interaction effects, such as *OA*, *DA*, *RA* and *RS*, are specified to be normally distributed with means set to zero and relatively small standard deviations, i.e.:

24 $$\begin{aligned} RS&\sim \textrm{Normal} \left( 0, 0.2^2\right) . \end{aligned}$$

The half-*t* prior for the standard deviation is considered a reasonable choice over conjugate gamma priors because it has a heavy tail and ensures that the data can dominate inference (Gelman, 2006). The half-normal prior is more restrictive of the larger values of standard deviations due to its lighter tail. In the cases when interactions capture similar effects, such as *RA* and $A_1$ in (17), the normal prior with a relatively small standard deviation hyperparameter constrains the region–age-specific deviations (*RA*) from the average age profile ($A_1$) to values closer to zero (on a logarithmic scale) and, thus, improves the parameter identification and stability of the algorithm. Constant *c* follows a weakly informative normal distribution $c\sim \textrm{Normal}(0,5)$ allowing it to capture the average observed rate (or count, for immigration). In the model for internal migration, the $\alpha _{ij}$ parameter has a standard normal distribution as a prior.

For the parameters in the time series models, we assume that the time effect for the first period $t = 1$ is zero, i.e. $\kappa _1 = 0$, which ensures the identifiability of the model parameters. For the parameters of the time effect models, we assume

25 $$\phi _{1} \sim {\text{Normal}}(0,2^{2} ),\;\;\phi _{2} \sim {\text{Normal}}(0.5,0.2^{2} ){\text{ }}\;1(0 \le \phi _{2} \le 1),$$

where $\phi _1$ denotes drift and $\phi _2$ the autoregressive parameters, respectively. This specification implies stationarity of the time series and cohort effects. The prior for the variance of the univariate time series model is half-standard normal. In the multivariate time series model, the prior for the correlation matrix $\Omega$ is of Lewandowski–Kurowicka–Joe form (denoted as LKJ; Lewandowski et al., 2009):

26 $$\begin{aligned} \mathbf {\Omega } \sim \textrm{LKJ} (2),\quad \sigma _s \sim \textrm{Normal}_+ (0, 1^2),\quad s\in \{M,F\}, \end{aligned}$$

where $\sigma _s$ is a diagonal element of matrix $\textbf{D}$ in (19). The $\textrm{LKJ}(\eta )$ prior with $\eta = 1$ is an equivalent of a uniform prior for correlation coefficients; when $\eta> 1$, the prior allows the correlation matrix to be shrunk towards the identity matrix. The specification $\eta = 2$ performed well in a simulation study and for the models presented in this paper.

Parameters of the bilinear term in the models capturing the changes over time in the age ($A_2$) and origin–destination ($OD_2$) profiles need to be constrained to ensure their identifiability. Here, we follow the specification outlined in Wiśniowski et al. (2015):

27 $$\begin{aligned} A_{2,(1:z-1)}&\sim \mathrm {Multivariate\ Normal}_{z-1} \left( \iota z^{-1}, z^{-2} \mathbf {\Psi }^{-1}\right) , \end{aligned}$$

28 $$\begin{aligned} A_{2,(z)}&=1-\sum _{i=1}^{z-1}A_{2,(i)} , \end{aligned}$$

where *z* is the last age group and length of $A_2$, $A_{2,(1:z-1)}$ denotes a vector of all but the last element of $A_2$, $\iota$ is a vector of ones (here, of $z-1$ length) and $\mathbf {\Psi }$ is a $(z-1) \times (z-1)$ precision matrix with 2 on the diagonal and 1 outside it. This specification allows the identification of parameters as long as the posterior values of $A_2$ are different from $z^{-1}$. The factor $z^{-2}$ that rescales matrix $\mathbf {\Psi }^{-1}$ ensures that the prior is sufficiently “wide” around its mean of $z^{-1}$ but not overly vague, which may lead to problems with parameter identification. It performed well in testing of the model using simulated data. In Appendix B, we present a full specification of the model and prior distributions for mortality.

## Application

### Data

To illustrate the proposed methods, we gathered annual data on mid-year Estimated Resident Population totals (1981–2016), annual birth registrations (1981–2016), annual death registrations (1993–2011), five-year internal migration transition counts based on Census data (1981–2016), annual immigration flows (1981–2016) and annual emigration flows (1981–2016). The immigration and emigration data refer to the “net overseas migration” numbers which are derived from passenger arrival and departure cards based on the “at least 12 months” definition prior to 2004 and the “12 out of 16 months” definition after 2004. The baseline population is from 2011, and we use available 2016 data to assess *ex post* predictive performance of our model. Note the models and forecast results in this paper do not account for the COVID-19 pandemic, which effectively closed the border to international migration—a substantial component of population growth in Australia. In the next section, we discuss some further modifications that could be included into the model framework to account for this major shock to the population system.

All of the above data were obtained by five-year age group, sex and state or territory from the Australian Bureau of Statistics. The eight states and territories of Australia are: New South Wales (NSW), Victoria (VIC), Queensland (QLD), South Australia (SA), Western Australia (WA), Tasmania (TAS), Northern Territory (NT) and Australian Capital Territory (ACT). The data obtained from the Australian Bureau of Statistics can be considered high quality with annual counts of fertility, mortality, immigration and emigration derived from administrative registers, and populations and internal migration from quinquennial censuses. Using high-quality time series data improves the accuracy of population forecasts. Low quality data, on the other hand, could potentially bias the forecasts or result in larger uncertainty. We assume all the data used in this paper are consistently measured across states and territories, noting that some states (e.g. New South Wales, Victoria and Queensland) are more populated than other states or territories (e.g. Tasmania, Australian Capital Territory, and Northern Territory), along with different population distributions and densities. If the analyses were extended to geographic areas below the state and territory level, we would expect some issues concerning data quality and sparseness to arise that would need to be addressed prior to including in the population forecast model. Finally, the multiregional population forecasting model has the advantage over single regional models by reducing bias and improving accuracy due to the inclusion of origin–destination probabilities of internal migration (Rogers, 1990).

### Results

All of the demographic component models, except for mortality, assumed stationary autoregressive process for the developments of the time effects, resulting in both the forecasts and their uncertainty remaining relatively stable during the forecasting period. In Appendix D and Figures 8-12, we present the assessment of the goodness of fit of the models to the data and provide brief summaries below.

**Fig. 2:**
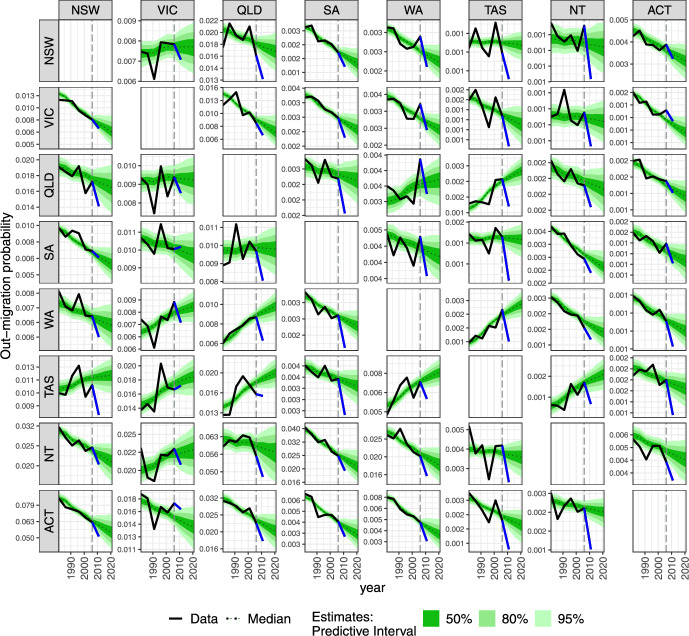
Origin (row)–destination (column) internal migration amongst states and territories of Australia, averaged over age groups and both sexes. The vertical dashed line denotes the cut-off point for fitting the internal migration model (1981–86 to 2006–11). States or territories: NSW—New South Wales, VIC—Victoria, QLD—Queensland, SA—South Australia, WA—Western Australia, TAS—Tasmania, NT—Northern Territory, ACT—Australian Capital Territory. Common logarithm is used for easier comparisons.

In Fig. 2, the forecasts of interregional out-migration probabilities (averaged over all age groups and both sexes and then logged) reflect the patterns that have been observed since the 1981–86 period. Internal migration that took place between 1981 and 1986 was recorded using transition approach in 1986, that is, by comparing residency at census date and five years before. Thus, the forecasts are based on six observations over time (quinquennial between 1986 and 2011) for each age, sex, origin and destination combination.

Despite the short time series, the model fits the data on probabilities reasonably well, smoothing out the small and zero probabilities observed in movements amongst smaller regions. We also note the overestimation of some of the 2011–16 out-migration probabilities which is due to the assumed autoregressive time series model that extrapolates the most recent observation and also borrows information across corridors. The largest model-based forecasts of out-migration probabilities are from the Northern Territory to other regions (especially Queensland) and from the Australian Capital Territory to New South Wales, for which in 2011–16 we forecast a median of 0.057 out-migration probability with a 95% Predictive Interval (PI) being (0.047, 0.069). The smallest internal migration is forecast into Tasmania and Northern Territory; for instance from New South Wales to Northern Territory the 95% PI is (0.0007, 0.0009). Our model also yields larger uncertainty for out-migration probabilities to and from the Northern Territory and to Victoria. The age and sex profiles of forecasted internal out-migration probabilities for the 2011–16 and 2021–26 period are presented in Figs. 13 and 14 in Appendix E.

**Fig. 3:**
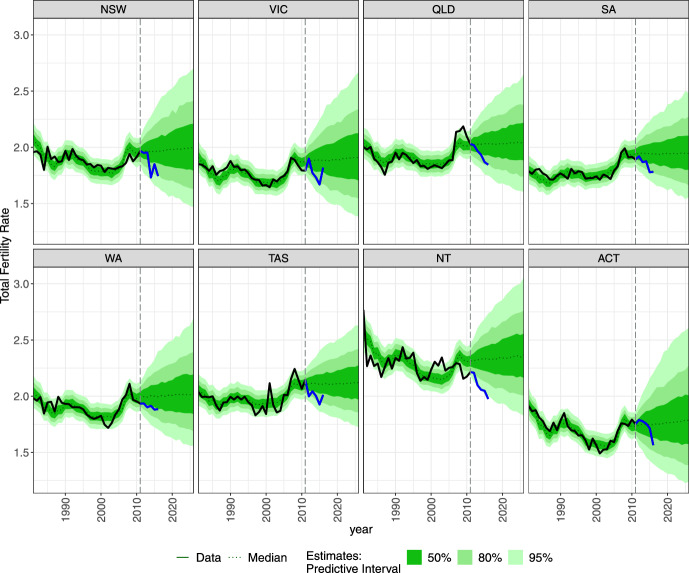
Forecasted total fertility rates for states and territories in Australia.

In Fig. 3, we present the forecasts of Total Fertility Rates (TFR) for the states and territories. The forecasted TFR reveal a stable pattern of medians being at similar levels as observed in 2011. For example, the median forecast for New South Wales in 2016 is around 2.0 (95% PI 1.64, 2.40); In 2026, this PI widens to (1.46, 2.74). The lowest median TFR for 2026 is forecast in Australian Capital Territory (1.80); the highest in Northern Territory (2.37). The TFR observed after 2011 showed a relatively sharp decline in all regions except for Victoria and Tasmania. Nevertheless, the observed out-of-sample data are within the Predictive Intervals of the model-based forecasts. In Fig. 15, we present the age-specific forecasts of fertility rates for all regions in 2016 and 2026.

**Fig. 4:**
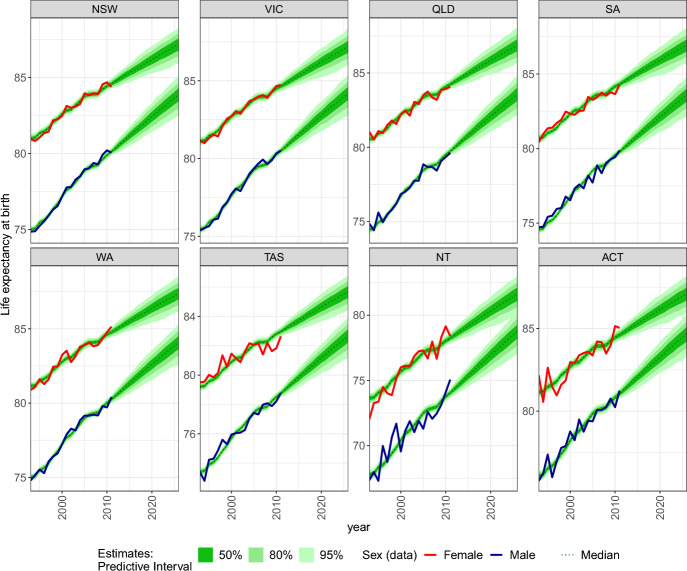
Forecasted life expectancies at birth for states and territories in Australia by sex (females = upper line, males = lower line).

Life expectancies at birth differ amongst regions as shown in Fig. 4. The highest life expectancy in 2026 is forecasted for Western Australia for females (median of 87.4 years) and Australian Capital Territory for males (median of 84.7 years). Our model produces considerably lower life expectancies for the Northern Territory (81.7 years for females and 78.8 years for males). The log-bilinear model fits and smooths the data reasonably well, only slightly overestimating the life expectancy in Tasmania in the second half of 2000s. The results also show that the male life expectancy is expected to increase at a faster rate than female life expectancy.

More sophisticated specifications of the model can ensure that life expectancies for both sexes converge to a common value (see, e.g., graduation in Dodd et al., 2018). In Fig. 16, we present age-specific mortality rates for states and territories in Australia in 2011 and 2026. We observe that the model fits the data well, especially when the observed data are relatively abundant (e.g. New South Wales and Victoria) and smooths out the cells with small and variable or zero observed deaths (e.g. Australian Capital Territory).

**Fig. 5:**
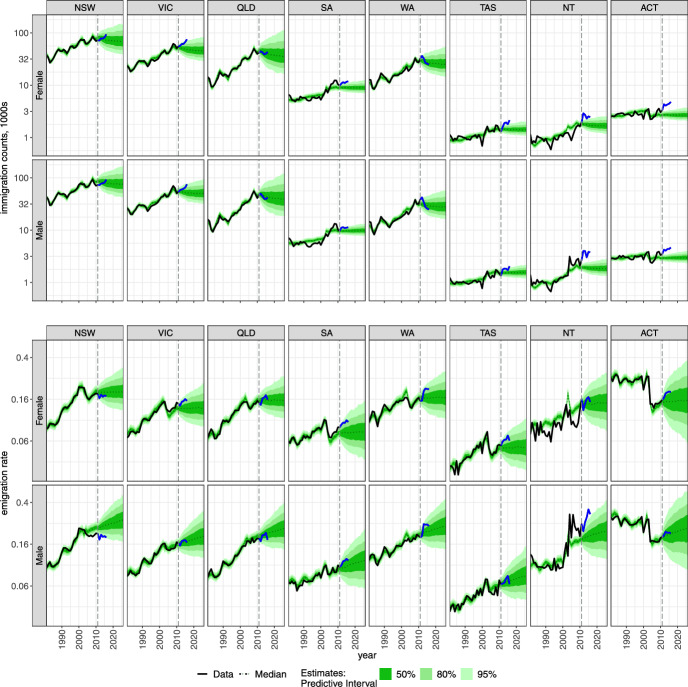
Forecasted immigration counts and emigration rates to states and territories in Australia and by sex. Note: common logarithm is used for easier comparisons.

The largest immigration counts (Fig. 5) are forecasted for the flows of males into New South Wales, reaching a 95% PI of around 43,000–170,000 annually in 2026, with a median of 75,000; slightly lower values are predicted for females. The lowest immigration is observed in the data and forecasted for Tasmania, with 95% PI being 1200–2200 immigrants in 2026 with a median of 1600 males.

The overall immigration trends remain relatively stable and even slightly decreasing in medians (see Fig. 18), unlike findings of Raymer and Wiśniowski (2018), where a moderate increase is forecasted for the total immigration to Australia. This can be partially explained by the slight underestimation of immigration to large regions such as Victoria and New South Wales (males only) for age groups 20–24 to 30–34 (see age profiles in Fig. 17). Further, we assume an autoregressive model rather than a random walk for the time effects of age and region profiles (Eq. 22). Nevertheless, we believe that a stationary autoregressive model provides sufficient description of uncertainty for the observed data and the forecast horizon under study. If desirable, other specifications of the time series models can easily be implemented.

The same log-bilinear model to forecast immigration counts was applied to emigration rates (Fig. 5). The resulting forecasts produced relatively stable patterns over time with slightly different median forecasts for males and females. Also similar to the immigration model, the temporal patterns after 2004 for Northern Territory were not well captured. The largest rates are observed in the data for the Australian Capital Territory though the change in the definition clearly affects the reported and forecasted emigration from that region. This justifies the inclusion of the parameter that captures the differences in definition specific to region ($R (t \ge 2004)$ in Eq. 21), rather than a global time-specific parameter. The age-specific rates are presented in Fig. 19.

The results from the multiregional population forecasting model are presented in Table 6 for males and females by state and territory in Australia. Our model-based forecasts for 2016 depict the observed 2016 data reasonably well, especially for the largest region New South Wales, Southern Australia and Northern Territory, and slightly under-predict the population of Victoria (which is just outside the 95% PI for males) and over-predict Tasmanian population by around 14,000 in total.

**Fig. 6:**
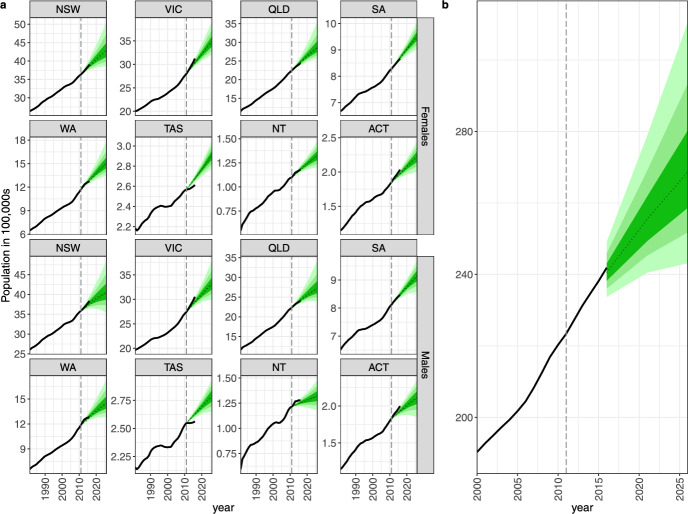
Forecasts of **a** population totals by sex for states and territories in Australia, **b** total population.

**Fig. 7:**
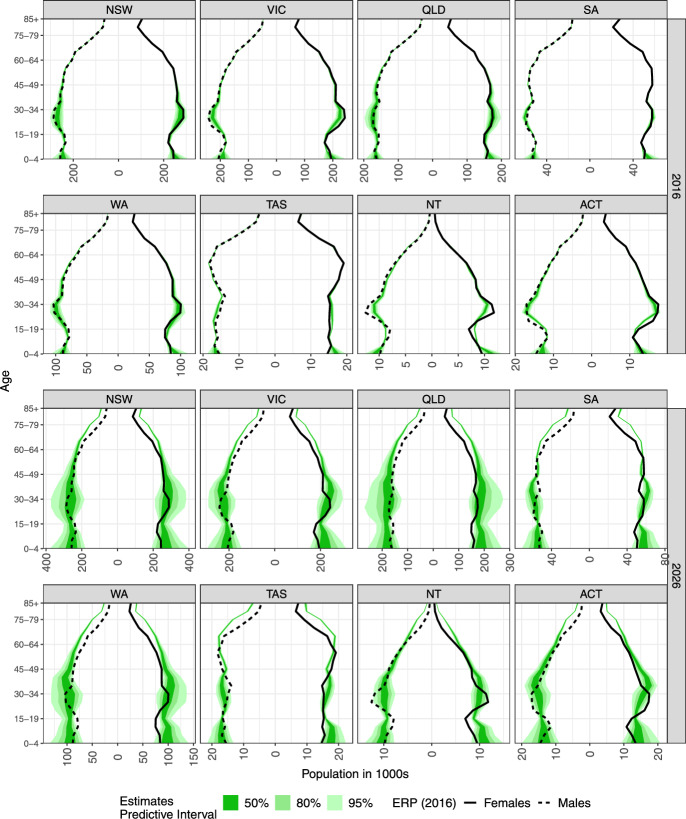
Forecasts of population pyramids for Australia by age and sex for 2016 (upper) and 2026 (lower). ERP is the Estimated Resident Population.

**Fig. 8:**
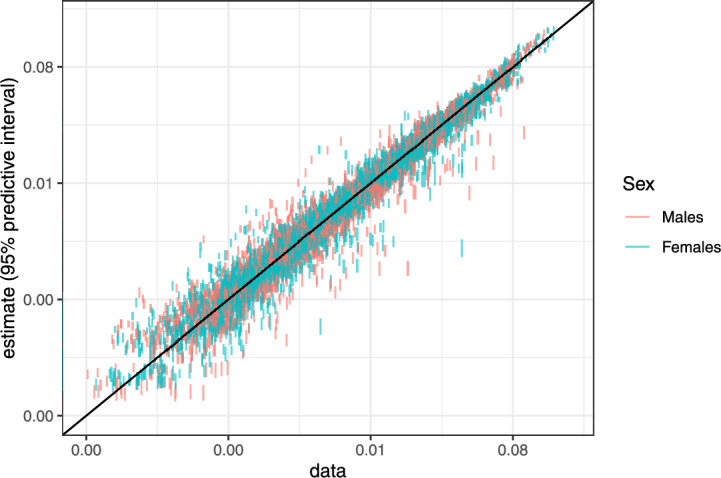
Comparison of the estimated and observed internal out-migration probabilities (age–sex–origin–destination–time-specific). Note: Data refer to the five-year transition periods 1981/86-2006/11.

**Fig. 9:**
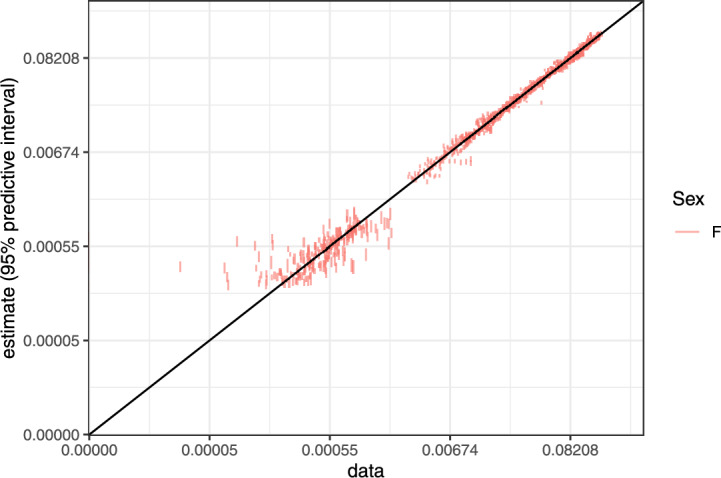
Comparison of the estimated and observed fertility rates (age–sex–region–time-specific). Note: Data refer to years 1981–2011.

**Fig. 10:**
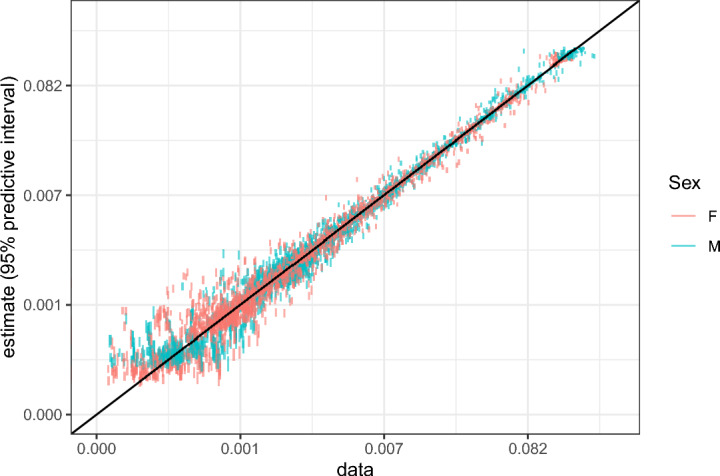
Comparison of the estimated and observed mortality rates (age–sex–region–time-specific). Note: Data refer to years 1993–2011.

**Fig. 11:**
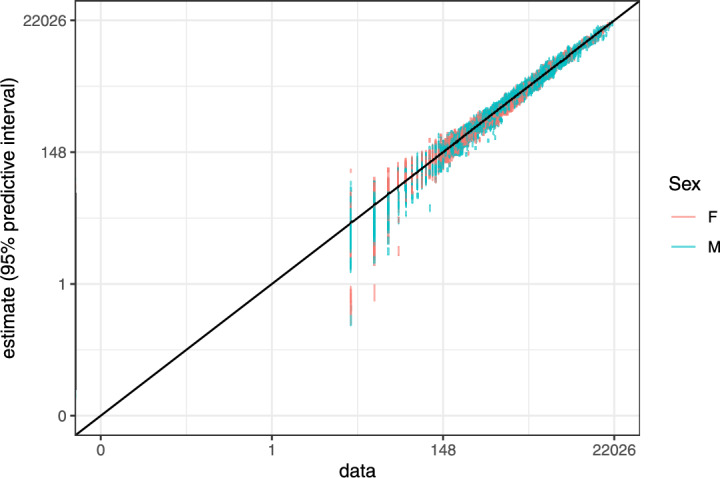
Comparison of the estimated and observed immigration counts (age–sex–region–time-specific). Note: Data refer to years 1981–2011.

**Fig. 12:**
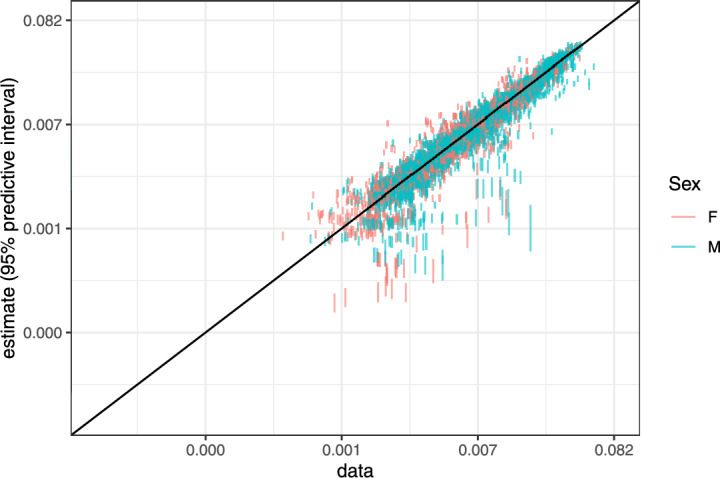
Comparison of the estimated and observed emigration rates (age–sex–region–time-specific). Note: Data refer to years 1981–2011.

**Fig. 13:**
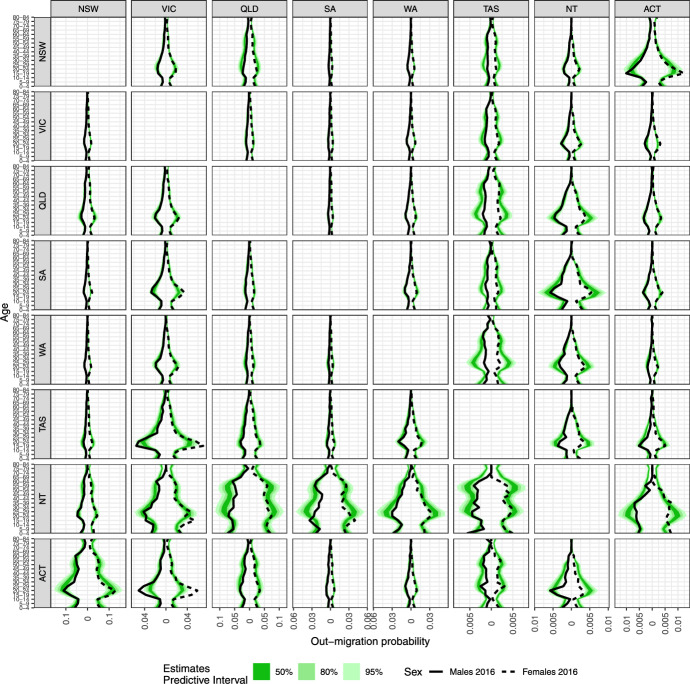
Age–sex profiles of observed and forecasted out-migration probabilities by state and territory of Australia: 2011/16. Note: Data and forecasts refer to the five-year transition period 2011–2016. Model was fitted using 1981/86-2006/11 data.

**Fig. 14:**
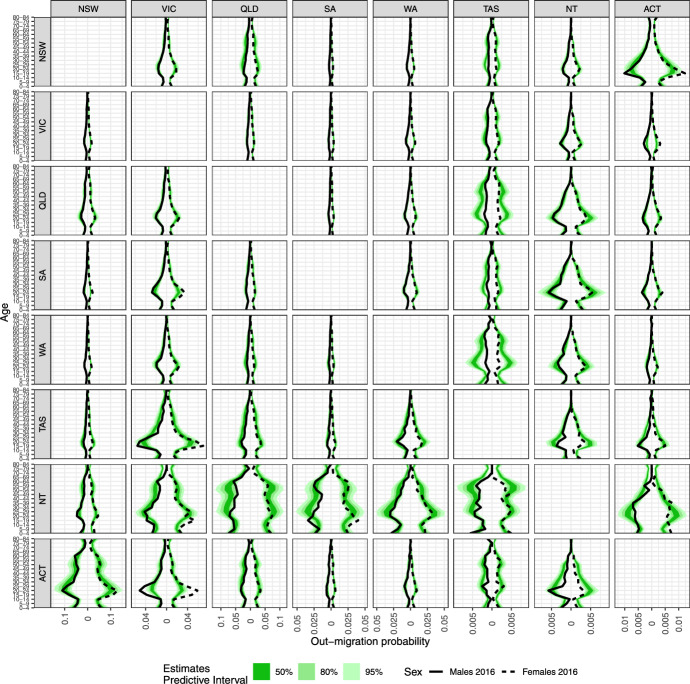
Age–sex profiles of forecasted out-migration probabilities by state and territory of Australia: 2021/26. Note: data (black lines) refer to the transition period 2011–2016; forecasts refer to the transition period 2021–2026.

The population forecasts for the regional and total populations are presented in Figs. 6a and b, respectively. When we decompose the forecasts from Fig. 6 into age profiles in 2016 and 2026 (Fig. 7), we note that the differences, for example, in Victoria, are driven by forecasts for age groups 20–34, which are slightly under-predicted. Changes to the sizes of these age groups are most likely driven by immigration (as explained earlier and in Fig. 17). The other differences are visible for much smaller populations of Northern Territory and Australian Capital Territory. Such populations are inherently more difficult to predict as a relatively small increase or decrease in, say, immigration, may lead to large relative changes in specific age–sex groups. The median forecast for a total population in Australia in 2016 is 24,056 thousand, which is 131 thousand smaller than the observed 24,186 thousand. The observed value also sits comfortably within the predictive interquartile range (23,761; 24,280) thousand, and it is the $64{\textrm{th}}$ percentile of the posterior distribution of the 2016 total population.

**Fig. 15:**
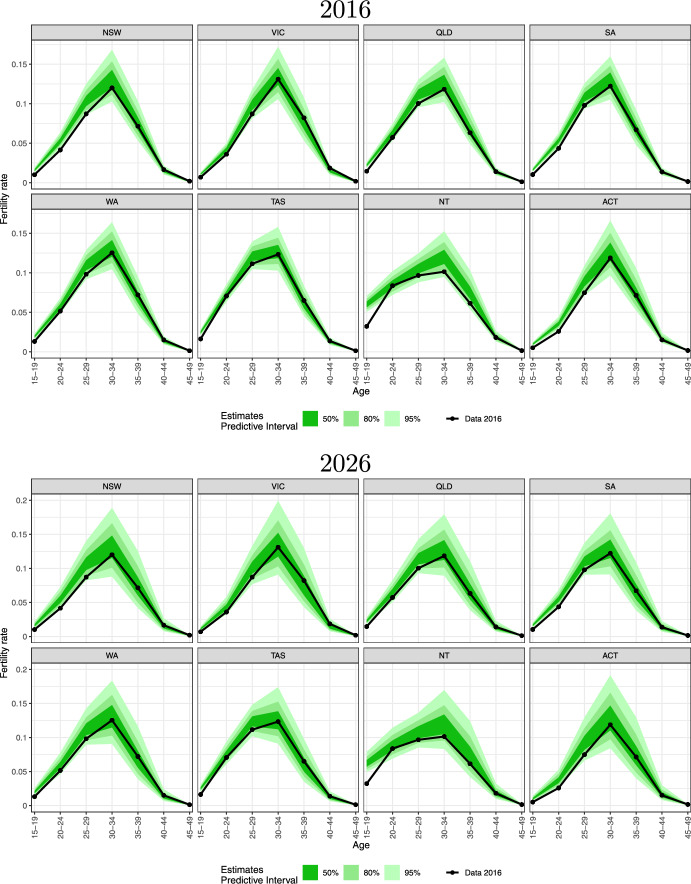
Age profiles of forecasted fertility rates by state or territory in Australia: 2016 and 2026. Note: model was fitted using 1981–2011 data.

**Fig. 16:**
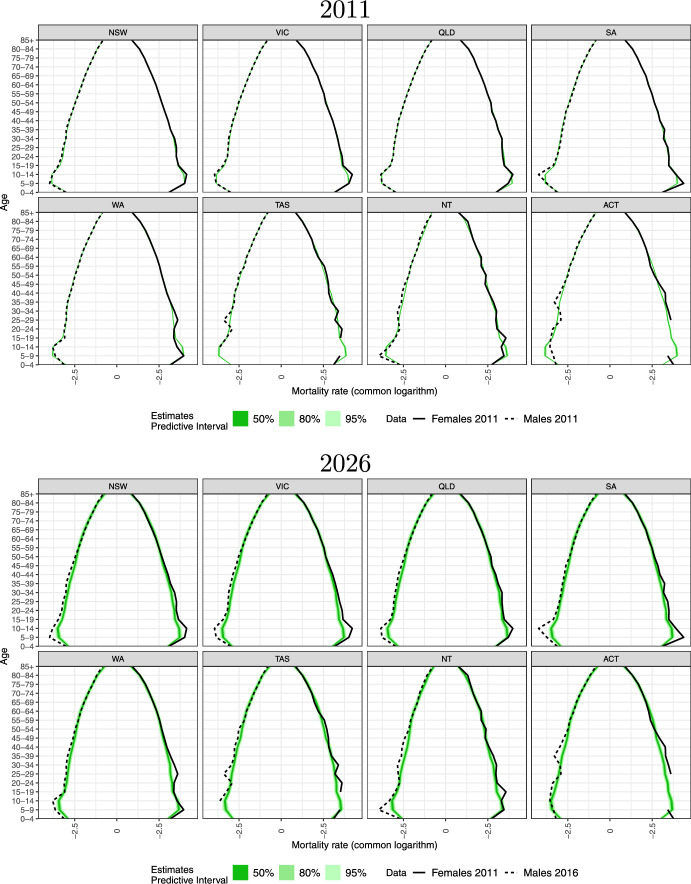
Age profiles of mortality rates (common logarithm) by state or territory in Australia: 2011 (model fit) and 2026 (forecasts). Note: model was fitted using 1981–2011 data.

**Fig. 17:**
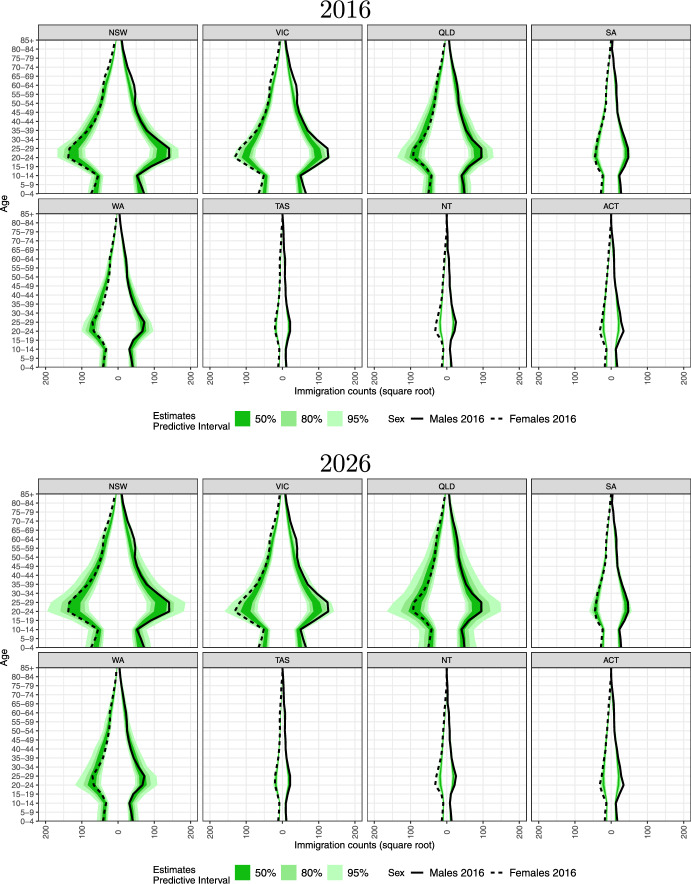
Age profiles of immigration to states and territories in Australia: 2016 and 2026. Note: model was fitted using 1981–2011 data.

**Fig. 18:**
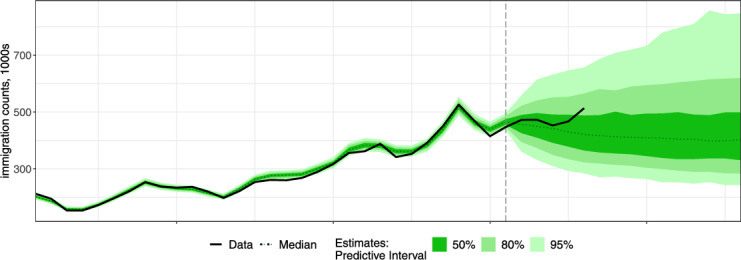
Total immigration to Australia as forecasted by the log-bilinar model. Note: model was fitted using 1981–2011 data.

**Fig. 19:**
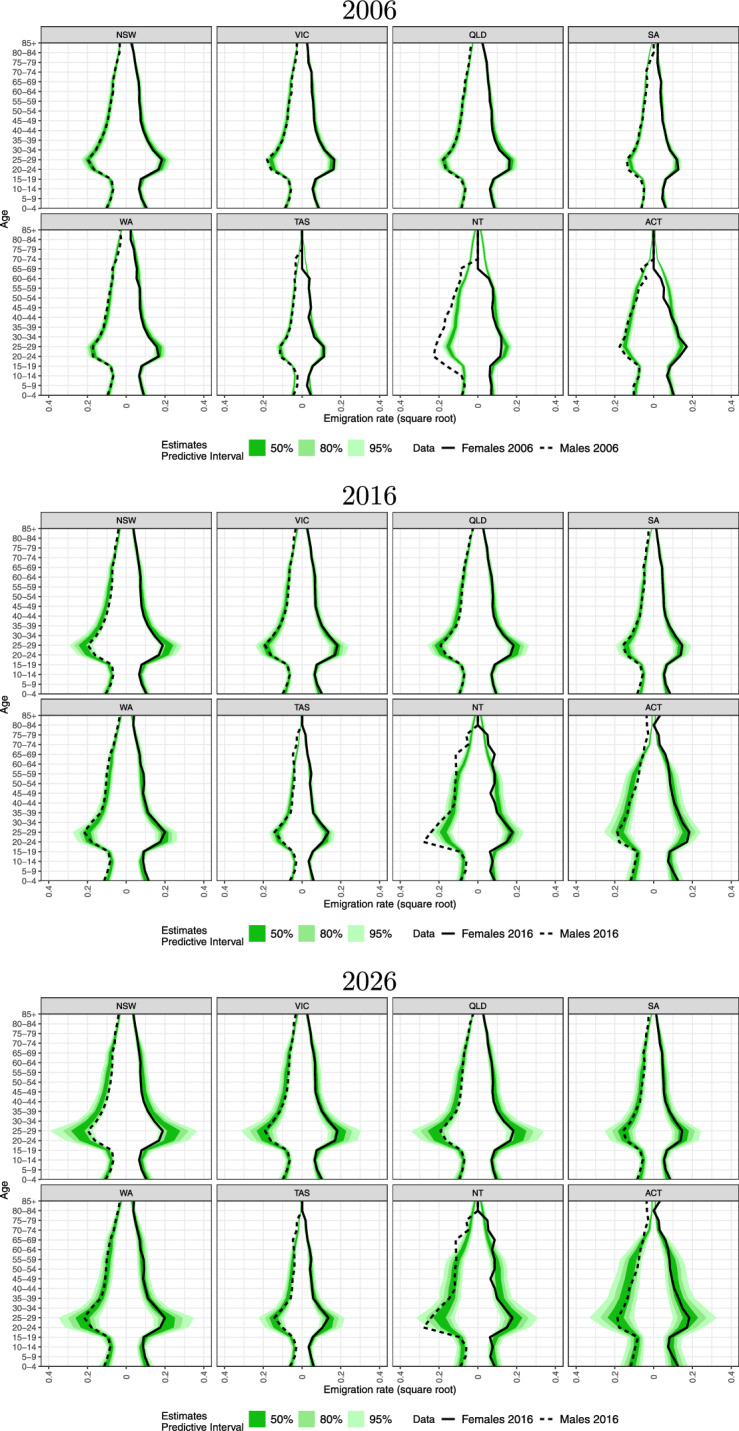
Age profiles of emigration from states and territories in Australia: 2016 and 2026. Note: model was fitted using 1981–2011 data.

The other differences between observed and estimated profiles arise in the youngest age groups. The main driver is an over-optimistic forecast of fertility, based on the data up till 2011. Over the following 2012–2016, the TFR drops rather dramatically. However, as mentioned above, the fertility forecasts acknowledge that by relatively wide predictive intervals, as depicted in Figs. 3 and 15.

## Conclusions

There are several contributions that this research provides. First, we have produced a modelling framework for probabilistic forecasting of subnational populations by age and sex, which provides inputs to a multiregional cohort-component projection model. The framework relies on bilinear models, such as Lee and Carter (1992) model used for mortality forecasting, combined with log-linear models typically used for analysing contingency tables. We demonstrated that these two models can be combined to reduce the large number of dimensions required for forecasting multiple characteristics of population. Second, we have developed and extended methods for estimating and forecasting the subnational components of population change over time, making best use of available data up to 2016 and our knowledge about them. Third, the forecasts include measures of uncertainty for all desired characteristics of the population and population change over time. Finally, we have demonstrated how the results from the models provide a more in-depth understanding of future population change by using data for states and territories in Australia.

We found that relatively small differences in the age profile of international migration disaggregated by regions can have relatively large influence on the subnational population forecasts. This finding confirms the importance and need for describing uncertainty of the international migration, as well as the role of international migration in shaping population structure of countries such as Australia. This effect is especially important in regions with small populations, as a relatively small, in absolute terms, change of population in a given age–sex group may be lead to large changes in relation to that population, which may result in large forecast errors.

This modelling framework can be extended in various ways. First, more complex models can be employed for all demographic components, in particular for mortality, fertility and international migration. Second, further work is required to deal with small areas where the incidence of demographic events is very low and measurement errors more likely to lead to biases, resulting in many zero counts and, subsequently, irregular age and sex patterns. The framework can be extended to incorporate the uncertainty about future counts, for example, by generating forecasts of counts of population components and imputing them directly into a population balancing equation (Bryant & Zhang, 2018). However, Yu et al. (2023) found that their forecasts for very small populations with uncertainty derived from rates performed “surprisingly” well and that Poisson variability may not be needed.

The main limitation of the current framework and its implementation is that it can be computationally expensive, especially when one would like to, for instance, extend the model to handle more characteristics of populations, test multiple models specifications, when large origin–destination migration data are available or when analysing data by single year of age. In our illustration, we overcame this limitation by testing simplified log-linear models (without bilinear component) with maximum likelihood method (Appendix C). Using integrated nested Laplace approximation (INLA) (Osgood-Zimmerman & Wakefield, 2023) method for deriving posterior distributions may bring efficiency gains. Computational difficulties will also have to be overcome in handling large arrays of data containing low and zero counts of events. Smoothing techniques that borrow information across space and time may provide a solution. To further reduce demand for computing time, the assumption about the sampling distribution of the data can be simplified into a normal model for logged or power-transformed rates of population events, rather than Poisson normal model with offset. Also, main effects in the multiplicative components sub-model may be used to model the rates instead of two- or more-way interactions. However, assumptions that simplify the models may lead to a poorer fit to the data and produce less accurate and/or biased forecasts. In principle, other distributional assumptions can be tested, for example, negative binomial model that has a parameter controlling under- or over-dispersion in the data, though we expect this to be more computationally intensive. Similarly, the internal out-migration probabilities could also be modelled by assuming a logistic model (Willekens & Baydar, 1986), but in situations with very small probabilities this may be computationally problematic.

The other important limitation of the proposed framework is that it requires detailed input data in form of contingency tables for all characteristics one would like to forecast. Time series of such detailed data may not be available, especially in countries with less developed statistical governance that leads to inadequate or missing data. Future work could see extensions of our framework to allow imputing missing characteristics, for example, by integrating data from multiple sources and fitting detailed cross-tabulations to known margins (e.g. Shen et al., 2024), or disaggregating margins by using multinomial models (e.g. Wiśniowski et al., 2016).

An excellent fit of a complex model to the existing data does not guarantee forecasts to be better than based on simpler models. Thus, more research is needed on the inclusion of expert-based information in the demographic component models, which may be especially relevant in the cases where data are limited, for example, only short time series are available, new policies are introduced, or if constraints are required to keep the quantities of interest, such as life expectancies or total fertility rates, within realistic bounds. One could also use informative expert-elicited priors to constrain, for instance, the average changes of mortality in the long term or for specific periods, if such were desired. This is particularly relevant in assessing the uncertainty of population change that occurred during the COVID-19 pandemic. While it had relatively minor impacts on mortality (Roser et al., 2020), it led to a near complete closure of the Australian border to international travel and migration. There were also periodic state and territory border closures within the country, which would have affected internal migration. While we wait for data to understand the long-term impacts of the pandemic on the Australian population, information could be elicited from experts and incorporated in the model in a form of prior distributions for model parameters (e.g. by imposing specific trajectories such as in Bijak and Wiśniowski, 2010), or distributions of future values of demographic components (Wiśniowski et al., 2014).

## Data Availability

The data used in the study are publicly available through the Australian Bureau of Statistics website. The data and code are made publicly available through a repository at 10.5281/zenodo.14680112.

## Appendix A: Multiregional Projection Model-Movements Approach

Projections for the vector of population aged $x+5$ in year $(t+5)$, denoted by $K_{x+5}(t+5)$, are made using the following equations:

A1 $$\begin{aligned} K_{0}\left( t+5\right)&=\frac{5}{2}\textbf{S}_{-5}\sum _{x=\alpha }^{\beta }\left( B_{x}+\textbf{S}_{x}B_{x+5}\right) \left( K_{x}\left( t\right) +\frac{1}{2}G_{x}\left( t\right) \right) +\frac{1}{2}G_{0}\left( t\right) , \end{aligned}$$

A2 $$\begin{aligned} K_{x+5}\left( t+5\right)&=\textbf{S}_{x}\left( K_{x}\left( t\right) +\frac{1}{2}G_{x}\left( t\right) \right) +\frac{1}{2}G_{x+1}\left( t\right) \end{aligned}$$

where *B* denotes fertility rates for all *R* regions, $\alpha$ and $\beta$ denote the first and last reproductive age groups, respectively (i.e. 15–19 years and 45–49 years) and *G* denotes a vector of immigration during entire 5-year period. Births $K_0$ are then split between males and females using the male birth-to-female birth ratio of 1.05–1.00. The matrix $\textbf{S}$ represents survivorship proportions that are computed using approximations derived by Rogers and Ledent (1976) and Ledent (1978, pp. 48–9):

A3 $$\begin{aligned} \textbf{S}_{-5}&=\left( \textbf{I}+\frac{5}{2}\textbf{M}_{0}\right) ^{-1} \end{aligned}$$

A4 $$\begin{aligned} \textbf{S}_{x}&=\left( \textbf{I}+\frac{5}{2}\textbf{M}_{x+5}\right) ^{-1}\left( \textbf{I}-\frac{5}{2}\textbf{M}_{x}\right) \mathrm {\ for\ } x = 0, 5, 10, {\ldots }, 5(\textit{z}-2),\end{aligned}$$

A5 $$\begin{aligned} \textbf{S}_{5(z-1)}&=\left( 5\textbf{M}_{5z}\right) ^{-1}\left( \textbf{I}+\frac{5}{2}\textbf{M}_{5(z-1)}\right) \end{aligned}$$

where $\textbf{I}$ denotes identity matrix, $\textbf{M}_x$ is a matrix containing mortality rates ($d_{xr}$), emigration rates ($e_{xr}$) and out-migration rates ($m_{x}^{ij}$) as estimated by models described in Sect. 3.^8^ The key difference in this model is that it includes out-migration rates exposed to the mid-year population $K_{ixst}$. The $\textbf{M}_x$ matrix is specified as

A6 $$\begin{aligned} \textbf{M}_{x}=&\left[ \begin{array}{cccc} d_{x}^{1 }+e_{x}^{1}+\displaystyle \sum _{j\ne i}m_{x}^{1j} & -m_{x}^{21} & \cdots & -m_{x}^{R1}\\ -m_{x}^{12} & d_{x}^{2D }+e_{x}^{2}+\displaystyle \sum _{j\ne i}m_{x}^{2j} & \cdots & -m_{x}^{R2}\\ \vdots & \vdots & & \vdots \\ -m_{x}^{1R} & -m_{x}^{2R} & \cdots & d_{x}^{R }+e_{x}^{R}+\displaystyle \sum _{j\ne i}m_{x}^{Rj} \end{array}\right] . \end{aligned}$$

## Appendix B: Specification of the Prior Distributions in the Mortality Model

The model is specified for the counts of deaths $Y_{rxst}$:

B7 $$\begin{aligned} Y_{rxst}&\sim \textrm{Poisson}(\mu _{rxst} K_{rxst}), \end{aligned}$$

with the log-expectation of the region- (*r*), age- (*x*), sex- (*s*) and time- (*t*) specific death rate

B8 $$\begin{aligned} \log \mu _{rxst}&\sim \textrm{Normal}\left( c + RA + RS + AS_1 + AS_2 \kappa _{st}, \sigma ^2\right) . \end{aligned}$$

The time-specific effects $\kappa _{st}$ for males ($s=M$) and females ($s=F$) follow a vector random walk model with intercept parameters $\phi _{11}$ and $\phi _{12}$:

B9 $$\begin{aligned} \left( \begin{array}{c} \kappa _{Mt}\\ \kappa _{Ft} \end{array} \right)&\sim \mathrm {Multivariate\ Normal}\left[ \left( \begin{array}{c} \phi _{11}+\kappa _{M\ t-1}\\ \phi _{12}+\kappa _{F\ t-1} \end{array} \right) ,\mathbf {\Sigma _1}\right] ,\qquad t=1,\ldots ,T. \end{aligned}$$

We assume that $$\left( \begin{array}{c} \kappa _{Mt}\\ \kappa _{Ft} \end{array} \right) =\left( \begin{array}{c} 0\\ 0 \end{array}\right)$$.

The priors of the log-linear model parameters are as follows:

B10 $$\begin{aligned} \sigma&\sim \textrm{t}_+ (2.5,0, 0.5), \end{aligned}$$

B11 $$\begin{aligned} c&\sim \textrm{Normal} \left( 0, 5^2\right) ,\end{aligned}$$

B12 $$\begin{aligned} RS&\sim \textrm{Normal} \left( 0, 0.2^2\right) ,\end{aligned}$$

B13 $$\begin{aligned} RA&\sim \textrm{Normal} \left( 0, 0.2^2\right) . \end{aligned}$$

The parameters of the bilinear (as in Lee–Carter model) model have the priors

B14 $$\begin{aligned} AS_1&\sim \textrm{Normal} (0, \sigma _{AS_1}^2),\end{aligned}$$

B15 $$\begin{aligned} \sigma _{AS_1}&\sim \textrm{t}_+ (2.5,0, 0.5), \end{aligned}$$

B16 $$\begin{aligned} A_{2,(1:z-1)}&\sim \mathrm {Multivariate\ Normal}_{z-1} \left( \iota z^{-1}, z^{-2} \mathbf {\Psi }^{-1}\right) ,\end{aligned}$$

B17 $$\begin{aligned} A_{2,(z)}&=1-\sum _{i=1}^{z-1}A_{2,(i)}, \end{aligned}$$

where $\iota$ denotes a vector of ones.

The priors for the time series model parameters are

B18 $$\begin{aligned} \phi _{11}&\sim \textrm{Normal} (0, 5),&\phi _{12} \sim \textrm{Normal} (0, 5), \end{aligned}$$

with the covariance matrix decomposed as $\mathbf {\Sigma _1}=\textbf{D}^{-1}\mathbf {\Omega } \textbf{D}^{-1}$, and the Lewandowski–Kurowicka–Joe prior being specified for $\mathbf {\Omega }$ and a half-normal prior for $\sigma _M$ and $\sigma _F$ – diagonal elements of (diagonal) matrix $\textbf{D}$:

B19 $$\begin{aligned} \mathbf {\Omega }&\sim \textrm{LKJ} (2),\end{aligned}$$

B20 $$\begin{aligned} \sigma _{\kappa s}&\sim \textrm{Normal}_+ (0, 1), s\in \{M,F\}. \end{aligned}$$

## Appendix C: Log-Linear Model Selection

Log-linear models are typically selected by using goodness of fit measures (statistics $X^2$ and $G^2$; cf. Kateri, 2014), information criteria and cross-validation. However, the number of possible models increases with the number of dimensions of the contingency table. There are two main approaches in testing goodness of fit of multiple models: *backward* and *forward* elimination. The *backward* elimination relies on estimating a saturated model (i.e. a model that fits data perfectly) and sequentially removing terms one at a time and testing goodness of fit or by using information criteria until test statistic changes significantly. The *forward* approach starts with main interactions (i.e. complete independence) and adds terms as long as they improve fit significantly. The cross-validation approach relies on subsetting the data into training and test samples and testing how well each model predicts the test sample.

In our work, we combine a *forward* elimination approach with cross-validation and demographic knowledge. Because the log-linear model with bilinear terms is computationally expensive, we first pre-select a candidate log-linear model and then transform it by replacing time-specific interactions with bilinear terms. We also rely, where possible, on a maximum of two-way (rather than three- or more-way) interactions. The preselection procedure is as follows:

1. Estimate a complete independence model, e.g. $A+S+R+T$ (where *A* denotes age, *S* – sex, *R* – region and *T* - time). For internal migration, region is replaced with *O* for origin and *D* for destination (and the two components are always added jointly); for fertility, sex *S* is dropped. As we assume that the key characteristic of a population component is age structure and how it changes over time, we test a model with *AT* interaction, $A+S+R+T+AT$. It is safe to assume that this interaction will almost always significantly improve the fit of the model to the data.
2. The second characteristic is age–sex structure; thus, we add an *AS* interaction, $A+S+R+T+AT+AS$.
3. Choose the best and parsimonious from the above models and add region-specific interactions that capture potential differences in age and sex profiles between regions:
  - Region–age, *RA* (for internal migration we add *OA* and *DA*),
  - Region–sex, *RS* (for internal migration we add *OS* and *DS*),
  - Both *RA* and *RS*.
4. To the best and parsimonious model with region-specific interactions, test for age–sex–time interactions (*AST*) and add region–time and sex–time interactions:
  - Age–sex–time, *AST* (for internal migration this step is tested last and for fertility this is not relevant),
  - Region–time, *RT* (for internal migration we add *ODT*; it is based on the literature that suggests the *OD* term is significant for this component),
  - Sex–time, *ST* (not needed if *AST* considerably improves fit),
  - Both *RT* and *ST*.
5. Finally, choose the best and parsimonious model in 4 and add a three-way interaction *AST*, if it is not in the model yet. This term, in a bilinear model, reflects that age profiles change over time separately for males and females. From demographic perspective, this is expected to be present in mortality and potentially in international and internal migration.

At each stage, we evaluate the goodness of fit through an RMSE of fitted and observed values. We also provide Bayes (Schwarz) Information Criteria (BIC) but tend not to rely on them as these tended to favour models with less parsimonious parameterisations as well as those that did not produce the lowest RMSE.

In the above approach, we give a preference to simpler log-linear structures that are able to capture key characteristics observed in demographic data. Should the approach be applied in other settings, model selection can be easily replaced by a purely data-driven one with multiple and complex bilinear models being tested before making a final selection to be transformed into a bilinear model.

Below, Tables 1, 2, 3, 4 and 5 present the BIC, RMSE and CV-RMSE for the models considered in the selection procedure.

**Table 1:** BIC, $\Delta$BIC and root mean square error of fitted values for log-linear models for internal migration

#	Model	BIC	$\Delta$BIC	RMSE
m1	A+S+O+D+T	799,853.70	640,596	205.06
m2	m1+AT	784,332.56	625,075	202.04
m3	m1+AS+AT	777,067.82	617,810	200.15
m4	m1+AS+OA+DA+AT	651,063.39	491,806	186.57
m5	m1+AS+OS+DS+AT	775,294.90	616,037	199.85
m6	m1+AS+OA+DA+OS+DS+AT	649,441.41	490,184	186.24
m7	m1+AS+OA+DA+AST	648,951.16	489,694	185.74
m8	m1+AS+OA+DA+AT+ODT	160,024.18	767	**62**.**12**
m9	m1+AS+OA+DA+AT+ST	650,303.92	491,046	186.26
m10	m1+AS+OA+DA+AT+ODT+ST	159,257.63	**0**	61.20

Bold denotes the lowest value

**Table 2:** BIC, $\Delta$BIC and root mean square error of fitted values for log-linear models for mortality

#	Model	BIC	$\Delta$BIC	RMSE
m1	A+S+R+T	71235.66	23645	119.82
m2	m1+AT	66386.46	18795	101.82
m3	m1+AS+AT	49667.26	2076	32.84
m4	m1+AS+AR+AT	47657.03	66	28.22
m5	m1+AS+SR+AT	49589.67	1999	31.83
m6	m1+AS+AR+SR+AT	47591.08	**0**	28.04
m7	m1+AR+AST	49439.42	1848	24.38
m8	m1+AR+SR+AST	49377.73	1787	24.14
m9	m1+AR+SR+AST+RT	49942.18	2351	**19**.**79**

Bold denotes the lowest value

**Table 3:** BIC, $\Delta$BIC and root mean square error of fitted values for log-linear models for fertility

#	Model	BIC	$\Delta$BIC	RMSE
m1	A+R+T	543,652.20	513,320.72	1589.13
m2	m1+AT	128,757.52	984,26.04	573.38
m3	m1+AR+AT	39,570.18	9238.70	300.06
m4	m1+AR+AT+RT	30,331.48	**0.00**	**153.15**

Bold denotes the lowest value

**Table 4:** BIC, $\Delta$BIC and root mean square error of fitted values for log-linear models for immigration

#	Model	BIC	$\Delta$BIC	RMSE
m1	A+S+R+T	636,786.38	470,821	434.44
m2	m1+AT	409,970.14	244,005	323.68
m3	m1+AS+AT	389,597.19	223,632	313.71
m4	m1+AS+AR+AT	331,248.94	165,283	286.12
m5	m1+AS+SR+AT	387,327.64	221,362	313.49
m6	m1+AS+AR+SR+AT	329,184.42	163,219	285.92
m7	m1+AR+AST	319,956.86	153,991	271.67
m8	m1+AR+SR+AST	317,892.33	151,927	271.42
m9	m1+AR+AST+RT	165,965.59	**0**	**128.67**

Bold denotes the lowest value

**Table 5:** BIC, $\Delta$BIC and root mean square error of fitted values for log-linear models for emigration

#	Model	BIC	$\Delta$BIC	RMSE
m1	A+S+R+T	350,727.57	219,349	210.97
m2	m1+AT	256,191.00	124,813	154.69
m3	m1+AS+AT	231,968.72	100,590	139.39
m4	m1+AS+AR+AT	190,700.98	59,323	124.22
m5	m1+AS+SR+AT	229,540.28	98,162	139.05
m6	m1+AS+AR+SR+AT	188,444.45	57,066	123.62
m7	m1+AR+AST	186,593.46	55,215	117.23
m8	m1+AR+SR+AST	184,403.14	53,025	116.39
m9	m1+AR+AST+RT	131,378.44	**0**	**62.18**

Bold denotes the lowest value

## Appendix D: Goodness of Fit of Models for Population Components

The goodness of fit of a Bayesian hierarchical model can be assessed by comparing the posterior predictive distribution (PPD) for the data with the observed values. Figures in this section (Figs. 8, 9, 10, 11 and 12) all present the PPDs along with the data to which they were fit. Next section presents PPDs of the forecasts for 2016 data that were not used in the estimation (Figs. 13, 14, 15, 16, 17, 18 and 19). The models fit the data reasonably well and are able to smooth irregularities in the data (e.g. when counts of events are zero). The models are better at predicting larger rates than smaller ones (the variability around the $45^{\circ }$ line is usually larger for smaller values). For immigration counts and especially emigration rates, the model tends to under-predict some of the rates. This is better visible in the age–sex-specific plots (Fig. 19, year 2006) where the observed rates for the teenage and working-age populations of females in Northern Territory are underestimated.

## Appendix E: Age and Sex Profiles of Demographic Components

Fig. 13, 14, 15 and 16; Table 6

**Table 6:** Forecasts of Australian population by region and sex for 2016 and 2026, rounded to the nearest thousand

	2011	2016	2016					2026				
	Data		p2.5	p25	p50	p75	p97.5	p2.5	p25	p50	p75	p97.5
*Females*												
NSW	3633	3899	3758	3838	3887	3934	4045	3861	4111	4295	4498	5059
VIC	2797	3122	2963	3010	3036	3064	3125	3207	3382	3492	3602	3901
QLD	2243	2442	2395	2446	2480	2514	2611	2578	2747	2867	3036	3544
SA	828	866	859	866	871	875	883	904	936	952	968	1006
WA	1168	1274	1253	1282	1297	1313	1350	1349	1444	1505	1579	1783
TAS	257	261	264	267	268	269	271	278	286	291	295	305
NT	110	118	115	117	119	120	122	120	128	133	137	147
ACT	185	203	192	196	198	200	203	200	215	223	230	245
*Males*												
NSW	3585	3834	3651	3738	3786	3837	3955	3552	3871	4049	4280	4851
VIC	2741	3051	2874	2927	2953	2982	3046	2999	3205	3323	3446	3790
QLD	2234	2403	2341	2402	2437	2475	2573	2401	2602	2728	2908	3421
SA	812	847	836	845	849	853	862	857	897	914	934	969
WA	1185	1282	1249	1280	1295	1312	1355	1283	1388	1452	1533	1727
TAS	255	256	260	262	263	264	266	265	274	279	284	293
NT	121	128	122	124	126	127	129	118	127	132	137	148
ACT	183	200	188	192	194	195	200	185	203	211	220	235

pX denotes Xth percentile of the posterior probability distribution

## Footnotes

1 Throughout this article, in- and out-migration refers to internal migration, whereas immigration and emigration to international migration.

2 To simplify notation, we drop subscripts denoting sex, region, time or origin and destination, unless explicitly specified. Matrices are in bold face. Also, when denoting time, $y(t)\equiv y_t$ and when denoting specific age groups, only the beginning age is included, e.g. age group 0-4 years is denoted by 0, 5-9 years is denoted by 5, and so on.

3 The representation of out-migration probabilities by a Poisson distribution with an offset may seem problematic because this framework does not constrain them to be always smaller than one. In principle, a logistic transformation could be employed to tackle this. However, in our illustration this is not of concern because of the data where we observe relatively small counts of out-migration events and denominators used in calculating transition probabilities are always large (cf. Sect. 5).

4 The 2.5 year in the subscript reflects the reference to mid-year population in the middle of the five-year period.

5 To simplify notation, when defining model $\mathcal {M}$ for a given population component we denote the dimension-specific effects, e.g. origin–destination effect by capital letters $OD_1$, which is equivalent to subscripts $OD_{1ij}$.

6 In general, this assumption can be relaxed by assuming $\alpha _{ij}\equiv 1$.

7 It follows notation used in Stan programming language, https://mc-stan.org/docs/functions-reference/student-t-distribution.html.
